# Training caregivers to screen for relapse among children who have recovered from severe acute malnutrition: study protocol for a feasibility trial

**DOI:** 10.1186/s40814-025-01752-z

**Published:** 2026-01-13

**Authors:** Clarisse Dah, Aimée Kimfuema, Mamadou Bountogo, Fanta Zerbo, Moussa Ouédraogo, Idrissa Kouanda, Ian Fetterman, Benjamin F. Arnold, Elodie Lebas, Ali Sié, Catherine E. Oldenburg

**Affiliations:** 1 Centre de Recherche en Santé de Nouna, Nouna, Burkina Faso; 2 Francis I Proctor Foundation, University of California, San Francisco, USA; 3 Department of Ophthalmology, University of California, San Francisco, USA; 4 Department of Epidemiology & Biostatistics, University of California, San Francisco, USA

**Keywords:** Severe acute malnutrition, Mid-upper arm circumference, Screening, Relapse, Wasting

## Abstract

**Background:**

Children with severe acute malnutrition (SAM) have a high risk of mortality and morbidity. After recovery from an initial episode of SAM, the risk of relapse can be high, although estimates vary across settings. Post-recovery surveillance for relapsed SAM in Burkina Faso consists of monthly clinic-based follow-up visits. However, adherence to the follow-up schedule can be variable, and children with missed surveillance visits may have delayed diagnoses of relapse. Here, we describe the protocol for a feasibility trial design to provide preliminary evidence to support the training of caregivers to screen for relapsed acute malnutrition using mid-upper arm circumference (MUAC) screening at home.

**Methods:**

This feasibility trial will enroll 200 caregiver-child dyads in which the child has recovered in the past month from an episode of SAM in Boromo, Burkina Faso. Eligible children had an initial episode of SAM that they recovered from per Burkinabè guidelines (weight-for-height *Z*-score, WHZ ≥ −2 and/or MUAC ≥ 12.5 cm, depending on the admission criteria). Caregiver-child dyads are randomized to either weekly screening using a standard MUAC tape plus standard of care follow-up or standard of care alone, which consists of monthly clinic-based screening for relapse for 3 months. Caregiver-child dyads are followed for 6 months. Primary feasibility endpoints include acceptability, time for training, enrollment potential and refusals, adherence to the follow-up protocol, and adherence to the screening protocol. Clinical endpoints, measured to inform the design of a full-scale trial, include the proportion of children relapsing, anthropometric measurements at 6 months, hospitalization, and vital status.

**Discussion:**

This feasibility trial will generate data to support the development and full-scale testing of an intervention to train caregivers to screen for relapsed acute malnutrition using MUAC.

**Trial registration:**

This trial is registered at clinicaltrials.gov (NCT05932992), first posted 27 June 2023.

## Background

Severe acute malnutrition (SAM) affects 19 million children every year, most of whom are in sub-Saharan Africa and South Asia [1]. SAM is defined as a mid-upper arm circumference (MUAC) of < 11.5 cm and/or weight-for-height *Z*-score (WHZ) < −3 among children aged 6 to 59 months. Uncomplicated SAM is treated on an outpatient basis, with children receiving a package of interventions including ready-to-use therapeutic food (RUTF), antibiotics, antiparasitics, and any missing vaccinations [2]. The major goal of these programs is to induce rapid, acute weight gain in children to improve the probability of survival during the acute episode. Children are followed weekly until nutritional recovery, typically for several weeks to months. While specific criteria for achieving recovery vary by country and context, children are typically considered to have recovered if they have met specific anthropometric thresholds, typically MUAC ≥ 12.5 cm and/or WHZ ≥ −2 with no edema, and the child has been in treatment for at least 4 weeks. Most interventions for SAM focus on improving the probability of nutritional recovery.

Relapse after recovery from SAM has been increasingly recognized as a contributor to poor outcomes. Evidence suggests a large degree of variation in the proportion of children who relapse after successful SAM treatment, with as many as 37% of successfully treated children relapsing up to 18 months after treatment [3]. Children with SAM with worse anthropometric deficits at program admission have worse outcomes compared to those with better anthropometric deficits [4]. Children who relapse with SAM are re-admitted to nutritional programs. Identifying children who have relapsed earlier may improve outcomes in these children.

Studies have previously shown that caregivers can be trained to detect acute malnutrition using MUAC measurements as well as community health workers [5]. In addition, the feasibility of caregivers screening for MUAC and danger signs of SAM has been demonstrated among children actively enrolled in outpatient nutritional programs for SAM [6]. However, a trial comparing a reduced follow-up schedule after admission to the program plus at-home surveillance using MUAC and training in clinical danger signs found lower nutritional recovery in the reduced follow-up schedule arm compared to the standard of care (weekly follow-up) arm [7]. In Burkina Faso, standard of care for follow-up following recovery from SAM includes monthly clinic visits for 3 months following program discharge. Burkina Faso has a high prevalence of SAM, with more than 100,000 cases among children under 5 years of age each year [8]. Here, we describe the protocol for a randomized controlled trial designed to evaluate the feasibility and acceptability of training caregivers to screen children who have recovered from SAM weekly for signs of acute malnutrition using a standard MUAC tape.

## Methods/design

### Aims and hypotheses

The overall objectives of this trial are to establish the feasibility of training caregivers to screen for relapse using standard MUAC tapes among children who have recovered from SAM. In addition, we aim to determine the burden of post-discharge relapse following recovery from an episode of SAM, to inform the design and feasibility of a full-scale trial of caregiver screening for the detection of relapse. We hypothesize that caregivers will find MUAC screening feasible and acceptable as demonstrated by the time for training, willingness to participate in training, enrollment, and follow-up in the trial, and accuracy of caregiver measurements.

### Ethics

The study was reviewed and approved by the Institutional Review Board at the University of California, San Francisco (Protocol 23–38829) and the Comite d’Ethique pour la Recherche en Santé in Ouagadougou, Burkina Faso (Protocol 2023-09-219). Written informed consent is obtained from the caregiver for both their participation and the participation of their child.

### Study design

This study is a 1:1 randomized feasibility trial in which caregivers of children who recovered from SAM in the past month are randomized to either training in weekly at-home screening using a standard MUAC tape for detection of relapsed SAM or standard of care, which includes monthly, clinic-based follow-up. Caregiver-child dyads are followed for 6 months from enrollment to assess feasibility, acceptability, accuracy, and anthropometric and other clinical endpoints.

### Setting

This study is being conducted in Boromo, Burkina Faso. Boromo district is in central Burkina Faso. This district experiences a large burden of SAM annually. This study is being implemented in 10 Centres de Santé et de Promotion Sociale (CSPS) which treat approximately 500 cases of SAM annually. CSPSs provide outpatient primary treatment, preventative care, and antenatal and maternity care, and host outpatient nutritional programs once a week in which they screen and provide care for uncomplicated SAM.

### Recruitment and eligibility criteria

Caregiver-child dyads are recruited based on nutritional program registers which record outcomes for all children who are treated as part of the outpatient nutritional program. Caregivers of children who have received outpatient treatment for SAM and who have recovered from their SAM episode are screened by a study nurse for eligibility in the present trial. Caregiver-child dyads are eligible to participate if the caregiver is at least 18 years of age, the child is between 6 and 59 months, the child has recovered from an episode of SAM per Burkinabè national guidelines (MUAC ≥ 12.5 cm and/or WHZ ≥ − 2, depending on which criterion the child was admitted to the nutritional program) in the past month, the family is planning to stay in the study area for 6 months to facilitate retention in the study, and with appropriate consent from the caregiver or guardian.

### Randomization and masking

Caregiver-child dyads are randomized on a 1:1 basis without stratification or blocking. Given the nature of the intervention, investigators, participants, and caregivers are not masked to their assigned treatment. To minimize contamination, each caregiver was given only a single MUAC tape for measuring their child, and multiple children from the same family were not enrolled. Nutrition program staff who evaluate children returning to the clinic for relapse are masked to the intervention assignment.

### Interventions

Caregivers who are randomized to the MUAC screening arm are trained by a study team member after enrollment in the trial. Each caregiver who is randomized to MUAC screening is given a standard color-coded MUAC tape. Caregivers receive a brief training in how to use and interpret the MUAC tape. Caregivers are instructed to measure the child’s MUAC weekly for 6 months and to return to the CSPS with their child for care if their child’s MUAC measurement is red (SAM, MUAC < 11.5 cm). In the standard of care arm, caregivers are instructed to bring their child in monthly for screening and nutritional counseling for three months, per Burkinabè national guidelines. Regardless of their anthropometric measurements, children who have recovered from SAM are enrolled in a moderate acute malnutrition (MAM) care program for three months. Children in the caregiver screening arm will also receive all the care packages provided in the standard of care. One study nurse per facility (*N* = 10 total) is responsible for implementing the intervention. Each nurse completed a 2-day didactic and practical training on study procedures prior to the start of the study. Intervention fidelity is ensured via regular supervisory visits from study investigators and supervisors to ensure the study protocol is followed.

### Participant timeline, procedures, and follow-up

**Fig. 1:**
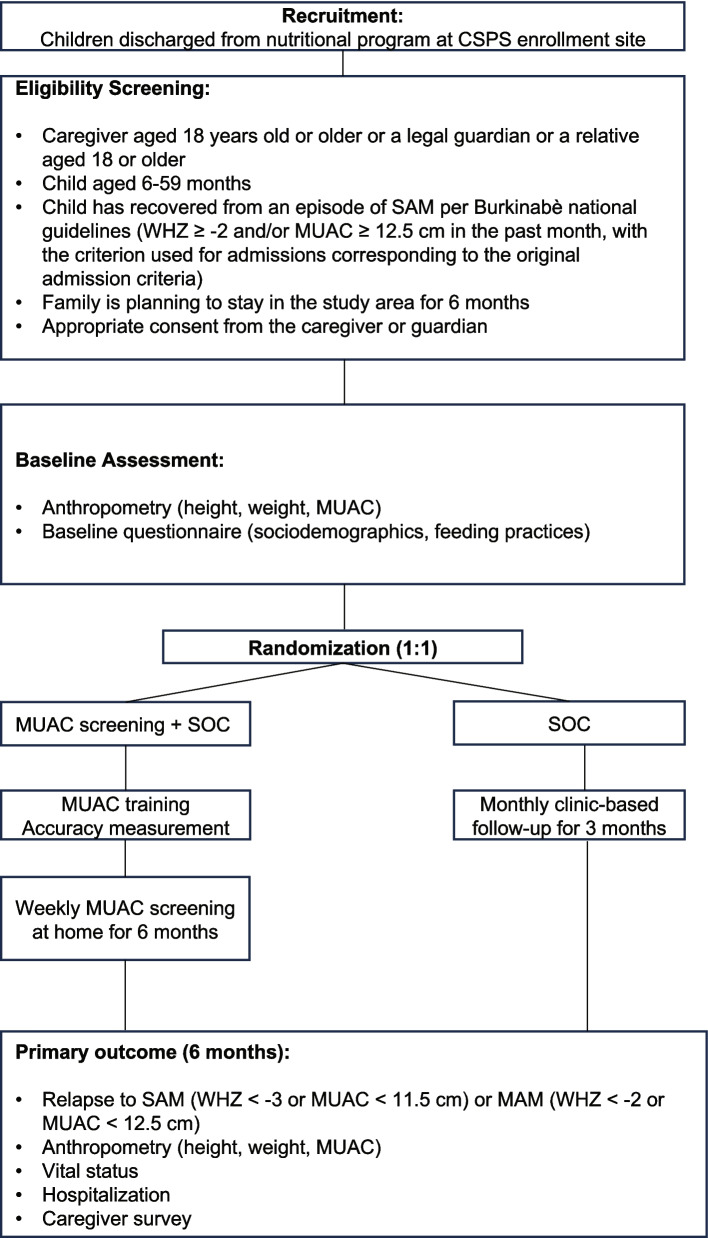
Participant flow diagram for the trial. Abbreviations: CSPS, Centre de Santé et de Promotion Sociale; SAM, severe acute malnutrition; WHZ, weight-for-height *Z*-score; MUAC, mid-upper arm circumference; SOC, standard of care

After enrollment, participants are followed monthly for 6 months. Per Burkinabè guidelines, caregivers are requested to bring their children back monthly for acute malnutrition screening and nutritional counseling. To understand adherence to the follow-up schedule, no efforts beyond those routinely implemented by the nutritional program to follow up children are employed. The primary endpoint of the trial is 6 months after enrollment (Fig. 1). While caregivers are requested to bring their child back to the clinic for this follow-up visit, phone calls to find the family and home visits are used in case the caregiver does not return to the clinic. At each study visit, anthropometric measurements are collected, including weight using a standing scale (Seca GmBH, Germany), height using a ShorrBoard (Weigh and Measure, LLC, Maryland, USA), and MUAC using a standard MUAC tape. At each follow-up visit, caregivers are asked about any care they sought for their child, reasons for seeking healthcare, and hospitalization.

### Feasibility and acceptability outcomes

The primary outcomes for the trial are focused on feasibility and acceptability. Feasibility endpoints focus on both the feasibility of conducting a large-scale trial that is powered for relevant clinical outcomes as well as feasibility of integration of caregiver screening into the existing health system. Acceptability focuses on acceptability of integration of caregiver screening from both the caregiver and clinic perspective.

- *Enrollment and follow-up*. We will assess the speed of enrollment and rate of refusal to enroll in the trial as an indicator of potential for enrollment in a full-scale trial. We will consider success to be enrollment of the target sample size in a 6-month period and refusal of < 10% of eligible participants. We will also assess follow-up at the 6-month primary endpoint visit and consider success to be retention of > 85% of participants at 6 months.
- Proportion of children who relapse. Assessment of the proportion of children who relapse over the 6-month period post-discharge will establish how frequently children relapse in this population, which will contribute to sample size calculations and justification for a full-scale trial. A low probability of relapse would indicate that interventions intended to reduce the risk of relapse are not warranted.
- Time to conduct MUAC training. To understand the additional burden on the existing healthcare system for training caregivers to screen for MUAC, the time training takes for each caregiver is measured during the enrollment visit. Extended time for training would be more difficult to implement in real-world nutritional programs. An acceptable time for training will be established via nutrition program interviews as part of the acceptability assessment, described below.
- *Accuracy of caregiver MUAC training*. At enrollment and 6 months, the accuracy of caregiver MUAC measurements against a gold standard (nutrition program staff) measure is collected. The caregiver and the nutrition program staff member each measure the MUAC of a child attending the clinic masked to each other’s measurements, categorized as “normal” (≥ 12.5 cm, green section of tape), “moderate acute malnutrition” (< 12.5 cm to ≥ 11.5 cm, yellow section of tape), or “severe acute malnutrition” (< 11.5 cm, red section of tape). Due to high levels of caregiver illiteracy, we will not collect numeric measurements. Concordance between color-based categories will be assessed to measure accuracy.
- *Adherence to the intervention*. Caregivers in the MUAC screening arm will be asked to report how often they screened their child at each follow-up visit. Infrequent caregiver screening would indicate that the intervention may not be acceptable or feasible. Caregivers are also asked to bring back the MUAC tapes at each visit, so the study team can confirm that they are still in possession of the caregiver and ensure that they are not damaged. Missing or damaged MUAC tapes are replaced and recorded.
- *Adherence to the follow-up schedule*. Standard of care in Burkina Faso includes monthly follow-up visits in the clinic to monitor for signs of relapse. By measuring how often children who have recovered return for scheduled follow-up in the standard of care arm, we aim to understand gaps in care. Low adherence to the follow-up schedule may suggest that additional interventions are needed, or a reduced follow-up schedule should be considered if relapse rates are low.
- *Caregiver acceptability*. Caregivers complete quantitative surveys related to their experiences screening children for relapse in the screening arm, including data on ease of use, frequency of screening, whether the MUAC tape was lost or stolen, and evaluation of caregiver-perceived importance of screening and whether this is a priority for them in relation to other demands in their lives. A subset of 20 randomly selected caregivers will be invited to participate in an in-depth individual qualitative interview, focusing on their experience with malnutrition screening, diagnosis, treatment, and relapse as well as their child’s general health, and their experiences related to screening. Interviews will be conducted by a study investigator with expertise in qualitative methods. Interviews will be transcribed, translated into English, and analyzed based on an inductive and deductive approach to identify themes guided by immersion crystallization.
- *Clinic acceptability*. Acceptability from the clinic perspective will be assessed via in-depth interviews with nutritional program staff and clinic leadership. These interviews will focus on the evaluation of MUAC training, ease of implementation, and general barriers perceived by the clinic in the delivery of outpatient treatment for SAM. As part of these interviews, we will evaluate the resources that were required to implement the screening intervention and what is acceptable for implementation from the clinic perspective, including personnel time and space and privacy for training.

### Clinical outcomes

In addition to acceptability and feasibility endpoints, a number of clinical endpoints are measured at each follow-up time point. Although the trial is not powered to detect a meaningful difference in clinical endpoints, these data will provide important preliminary data for the design of a full-scale trial should feasibility criteria be met.

- *Proportion of children relapsing*. The proportion of children who relapse will be compared between study arms. Relapse to SAM will be defined as MUAC < 11.5 cm and/or WHZ < −3. Children who relapse to SAM will be re-admitted to the nutritional program. These children will continue to be monitored in the study. We will also assess the proportion of children who relapse to MAM by arm, defined as MUAC < 12.5 cm and/or WHZ < −2.
- *Time to detection of relapse*. Time from enrollment to first relapse will be analyzed in both arms. The date of relapse will be defined as the earliest date the relapse was identified in the clinic, via a planned study visit or unscheduled clinic visit.
- *Anthropometric outcomes*. At all study visits, weight, height, and MUAC measurements will be collected. We will compare growth based on weight and height as well as anthropometric measures defined by weight-for-age *Z*-score (WAZ), height-for-age *Z*-score (HAZ), and WHZ, calculated based on 2006 WHO child growth guidelines [9].
- *Vital status*. Vital status is assessed at the 6-month visit, and children are classified as alive, died, or unknown. Families who do not return to the clinic for the 6-month study visit will be contacted via phone call and/or home visit to ascertain vital status.
- *Hospitalization*. At each study visit, caregivers will be asked about any health care sought for the child since their previous visit. Reasons for seeking health care, and if the child was hospitalized overnight, will be recorded. In addition, data from the clinics in which children are enrolled are extracted from clinic records, and hospital records are queried, and data recorded for any hospitalizations of children enrolled in the study. To facilitate linking children to clinic and hospital records, a unique study identification number is placed in each child’s government-issued health card (*carnet de santé*). Clinic and hospital staff are sensitized to record this information for children enrolled in the trial.

### Data collection and management

Data are collected electronically using a custom mobile data application (CommCare, Dimagi, Inc, Massachusetts, USA). Data are uploaded daily to a secure, cloud-based server that is password protected and Health Insurance Portability and Accountability Act (HIPAA) compliant. Data are collected on mobile phones that are password protected. All study team members underwent training to learn how to use the mobile devices and data collection application as well as best practices for data collection and confidentiality. Data collection is monitored on an ongoing basis via generation of twice-weekly reports to evaluate study progress, data collection quality, follow-up, and adverse events. Any concerns related to data quality or completeness are discussed with the data collection team, and refresher trainings and supervision visits are completed as needed.

### Sample size

A sample size of 200 caregiver-child dyads was chosen for logistical and resource reasons to provide data for feasibility outcomes and inform the sample size calculation for a future trial [10]. We anticipated we would be able to recruit this number of children who recovered from an episode of SAM during a single malnutrition season based on a previous study that enrolled children with SAM during one malnutrition season and measured the number that recovered [11]. The primary goal of this study is to establish the acceptability, feasibility, and accuracy of caregiver screening, and the trial is not powered for clinically meaningful differences in clinical outcomes.

### Statistical analysis plan

Acceptability and feasibility outcomes will be analyzed descriptively. For clinical outcomes, dichotomous outcomes, including the probability of relapse, we will compare the proportion of children with the outcome by study arm using a Fisher’s exact test. We will compare time to relapse using a log-rank test with a term for randomized treatment assignment. Anthropometric outcomes will be compared by arm with a term for randomized treatment arm and the baseline anthropometric measure, except for growth outcomes (weight gain in g/kg/day and height change in mm/day).

### Dissemination plan

Results of the pilot study will be shared with local stakeholders, including clinic leadership, as well as members of the Ministry of Health in Burkina Faso. Results will be presented nationally and internationally via presentations at conferences and publication in peer-reviewed journals.

## Discussion

Children with SAM are at high risk of mortality [12, 13], and interventions for SAM often focus on the acute phase of recovery immediately following admission to the nutritional program. Relapse to SAM or MAM following recovery from an episode of SAM is common, and these children may be at increased risk of mortality and morbidity due to repeated episodes of acute malnutrition [3, 14–17]. However, evidence-based interventions for prevention of relapse and post-discharge mortality are limited [18]. In the current trial, we aim to establish the feasibility and acceptability of training caregivers to screen for relapsed acute malnutrition using standard MUAC tapes. The 2023 WHO guidelines on prevention and management of acute malnutrition include nutritional counseling and education at the time of exit from the nutritional program after recovery [19]. If acceptable, feasible, and effective, inclusion of training in screening for relapse in caregiver screening at the time of exit may be an easily scalable intervention to reduce relapsed wasting in children under 5 years of age.

We anticipate that the current trial will provide critical feasibility data to inform both a full-scale trial and future interventions related to MUAC screening for relapsed SAM. Feasibility data will include both quantitative and qualitative measures of caregiver and clinic experiences with MUAC screening, and these data may inform changes to training or other protocols for MUAC screening interventions to be tested in a full-scale trial. In addition, data will provide critical inputs for sample size calculations. Data collected as part of this feasibility trial will help identify gaps in current post-recovery SAM surveillance, for example, by evaluating adherence to the follow-up protocol. This may lead to the development of new or additional interventions that could be integrated with MUAC screening to improve post-discharge surveillance for children who have recovered from an episode of SAM.

This feasibility trial is designed to provide preliminary evidence supporting larger-scale evaluation of MUAC-based screening interventions for caregivers of children who have recovered from SAM. The study is not powered to detect potentially meaningful clinical differences between children whose caregivers have been randomized to the MUAC screening arm compared to standard of care. In addition, this study does not evaluate a reduced follow-up schedule post-recovery from SAM. Although a reduced follow-up schedule may lead to worse outcomes during the acute phase [7], if MUAC screening with the standard follow-up schedule is feasible, future studies could consider evaluation of a reduced post-recovery follow-up schedule to reduce caregiver burden. Data from this trial would help inform such a study, for example, by establishing caregiver adherence to screening and follow-up. Finally, it is possible that caregivers will share MUAC tapes, which could introduce contamination, further limiting the study’s statistical power. However, we anticipate that the study will yield important data relating to implementation of this intervention, including information on MUAC tape sharing.

Overall, we anticipate that the results of this feasibility study will provide preliminary evidence of the integration of MUAC screening by caregivers into the post-recovery follow-up protocol for children who have recovered from an episode of SAM. This work is expected to support future evaluation of interventions for the prevention of relapse in this population.

### Trial status

The text of this manuscript refers to protocol version 1.4, 18 December 2023. Recruitment began on 1 December 2023 and is ongoing as of 31 January 2024. The trial is expected to continue through September 2024.

## Data Availability

Not applicable (no data reported).

## Abbreviations

SAM: Severe acute malnutrition
MUAC: Mid-upper arm circumference
WHZ: Weight-for-height *Z*-score
RUTF: Ready-to-use therapeutic food
CSPS: Centre de Santé et de Promotion Sociale
HAZ: Height-for-age *Z*-score
WAZ: Weight-for-age *Z*-score
HIPAA: Health Insurance Portability and Accountability Act
WHO: World Health Organization
